# Compact Wideband Tapered Slot Antenna Using Fan-Shaped and Stepped Structures for Chipless Radio-Frequency-Identification Sensor Tag Applications

**DOI:** 10.3390/s24123835

**Published:** 2024-06-13

**Authors:** Junho Yeo, Jong-Ig Lee

**Affiliations:** 1 Department of Artificial Intelligence, Daegu University, 201 Daegudae-ro, Gyeongsan 38453, Republic of Korea; 2 Department of Electrical and Electronics Engineering, Dongseo University, Busan 47011, Republic of Korea; leeji@gdsu.dongseo.ac.kr

**Keywords:** compact wideband tapered slot antenna (TSA), miniaturization method, fan-shaped structures, stepped structures, chipless radio frequency identification (RFID)

## Abstract

In this paper, two kinds of miniaturization methods for designing a compact wideband tapered slot antenna (TSA) using either fan-shaped structures only or fan-shaped and stepped structures were proposed. First, a miniaturization method appending the fan-shaped structures, such as quarter circular slots (QCSs), half circular slots (HCSs), and half circular patches (HCPs), to the sides of the ground conductor for the TSA was investigated. The effects of appending the QCSs, HCSs, and HCPs sequentially on the input reflection coefficient and gain characteristics of the TSA were compared. The compact wideband TSA using the first miniaturization method showed the simulated frequency band for a voltage standing wave ratio (VSWR) less than 2 of 2.530–13.379 GHz (136.4%) with gain in the band ranging 3.1–6.9 dBi. Impedance bandwidth was increased by 29.7% and antenna size was reduced by 39.1%, compared to the conventional TSA. Second, the fan-shaped structures combined with the stepped structures (SSs) were added to the sides of the ground conductor to further miniaturize the TSA. The fan-shaped structures based on the HCSs and HCPs were appended to the ground conductor with the QCSs and SSs. The compact wideband TSA using the second miniaturization method had the simulated frequency band for a VSWR less than 2 of 2.313–13.805 GHz (142.6%) with gain in the band ranging 3.0–8.1 dBi. Impedance bandwidth was increased by 37.8% and antenna size was reduced by 45.9%, compared to the conventional TSA. Therefore, the increase in impedance bandwidth and the size reduction effect of the compact wideband TSA using the second miniaturization method were better compared to those using the first method. In addition, sidelobe levels at high frequencies decreased while gain at high frequencies increased. A prototype of the compact wideband TSA using the second miniaturization method was fabricated on an RF-35 substrate to validate the simulation results. The measured frequency band for a VSWR less than 2 was 2.320–13.745 GHz (142.2%) with measured gain ranging 3.1–7.9 dBi.

## 1. Introduction

As the era of the fourth industrial revolution progresses to realize a hyper-connected intelligent information society in which both people and objects are connected, and collected data are analyzed and utilized based on artificial intelligence, the demand of wideband wireless communication technology is extensively increasing in order to satisfy the need to support more users and to provide more information with higher data rates [1,2]. The design of a wideband antenna is necessary to support the wideband wireless communication technology [3]. Wideband antennas can be divided into omni-directional and directional antennas depending on radiation patterns or directivity of the antenna. The types of directional wideband antennas include Yagi-Uda antennas, quasi-Yagi antennas, log-periodic dipole array antennas, spiral antennas, tapered slot antennas (TSAs), horn antennas, and reflector antennas [4]. Among various directional wideband antennas, TSAs have been widely used because they are thin with a planar structure, are easy to manufacture, and have relatively high gain characteristics over a wide band [5]. The concept of the TSAs began in the late 1950s as a flared slot antenna with the slot width gradually increasing [6]. TSAs with an exponentially tapered slot were introduced by Gibson as the Vivaldi antenna in 1979 [7] and have been actively studied in the fields of ultra-wideband (UWB) communications, radar, and imaging. TSAs can be classified into constant width, linear, stepped, elliptical, exponential (Vivaldi), and double exponential, depending on the shape of the slot [5].

Compact wideband directional antennas are required in application fields, such as a UWB communication for wearable devices, UWB radar and imaging systems, wireless body area networks, and retransmission-based chipless radio frequency identification (RFID) systems [8,9,10]. Miniaturization methods to reduce the dimensions of the TSAs found in the literature can be classified as follows: an addition of the slots with various shapes to the sides of the ground conductor, an insertion of the corrugations to the sides of the ground conductor, and a modification of the shape of the tapered slot [11]. Firstly, the slots with various shapes, such as exponential, triangular, anti-spiral, quarter circular, half circular, eye-shaped, eye-shaped combined with a circle, modified exponential, fractal-shaped, and hook-shaped, were appended to the edges or sides of the ground conductor for the miniaturization of the TSAs. When the slots were etched on the sides of the ground conductor, the outer electrical length of the TSAs was increased, and this, in turn, moved the lower limit of the frequency band, which has a size-reduction effect. A bunny-ear-shaped TSA with an exponentially tapered ground conductor operating in the frequency range from 0.5 GHz to 18 GHz was proposed to reduce the size of the TSA [12]. A compact, linear TSA with a combination of a triangular slot on the left side and corrugations on the right side of the ground conductor was introduced to operate from 3.1 GHz to 11 GHz [13]. However, the frequency bandwidth was somewhat limited by the coplanar waveguide feed to a delay slotline transition. A compact sheep-horn-shaped TSA with a pair of anti-spiral-shaped structure loadings at the ends and two lumped resistors at the half path of the spirals covering a frequency band of 1.2–9.8 GHz was reported [14]. In this case, the gain of the TSA was not provided and the lumped resistors might reduce antenna gain. A pair of quarter circular slots and nonuniform corrugations were added on the sides of the ground conductor of an elliptical TSA to cover a frequency band of 3.1–10.6 GHz, but the antenna performance was similar to the TSA with the triangular slot and corrugations [15]. A miniaturized TSA with a combination of quarter circular slots and stepped structures for further size reduction was proposed to cover 2.35–11 GHz [16]. However, the frequency bandwidth might be extended by using curved structures instead of linear structures. A compact TSA using a pair of half circular slots operating in a frequency band of 4.5–50 GHz with large bandwidth was also reported, but the antenna width was quite large compared to other compact TSAs in the literature because of the circular patches appended to the upper part of the TSA [17]. Two pairs of eye-shaped slots were added on the sides of the ground conductor for designing a compact TSA with an operating frequency band ranging from 3 GHz to 12.6 GHz [18]. More pairs of slots can be used for size reduction if the ground conductor has enough space to add them. Resonant cavities consisting of an eye-shaped slot and a circular slot were introduced to the edges of the ground conductor to make a compact TSA covering 0.5–6 GHz [19]. However, gain at low-frequency regions was less than 1 dBi, so a tradeoff between the size reduction and lowest gain in the band should be considered. A miniaturized dual-layer TSA with a pair of modified exponential slots operating in the frequency range from 2.5 GHz to 11 GHz was reported, but the antenna height was doubled due to the dual-layer structure [20]. A comparison of performance and dimensions based on using either exponential slots or circular slots might need to be investigated. The slots using the third generation of Koch fractal curve with a combination of circular slots were used to make a compact TSA operating from 4.87 GHz to 11 GHz, but the Koch fractal curve-based slots would be very difficult to design in practice because of their complex structure and time-consuming geometry calculation [21]. Hook-shaped slots were appended to the sides of the ground conductor for a compact linear TSA with a frequency band of 2.83–11.31 GHz [22]. In this case, a new design using a different taper shape and slots using the curve-based structures instead of straight line-based structures might need to be investigated for further miniaturization. Four sequentially rotated Vivaldi elements were bent into the antenna aperture for the miniaturization of the wideband directional circularly polarized antenna operating in the frequency band from 1.6 GHz to 2.9 GHz using a 1-to-4-integrated feeding network with identical amplitudes and an orthogonal phase [23]. However, the antenna height was quite large due to the bent Vivaldi structures. Four cascaded circular cavity structures based on several circular holes in tandem with different curvatures were appended on the sides of the ground conductor of a Vivaldi antenna to cover the 0.45–10 GHz frequency band, but gain at low-frequency regions was less than 0 dBi and the antenna size was very large [24].

Secondly, corrugations were inserted on the sides of the ground conductor to miniaturize the TSAs. Corrugations are a periodic arrangement of uniform or nonuniform slits (narrow rectangular, triangular, trapezoidal or arbitrary-shaped slots). They can also be used for different purposes, such as gain enhancement and radiation pattern improvement. A compact coplanar waveguide-fed TSA using three pairs of trapezoidal slots was proposed to operate on a frequency band from 3 GHz to 11.4 GHz [25]. Effects of adding more pairs of the slots on the size reduction and gain enhancement need to be investigated systematically. The miniaturization performance of rectangular, cosine, and sawtooth (triangular) corrugations added to the TSA with multi-section binomial transformers were compared for UWB applications [26]. When 42 pairs of corrugations with three different types were appended, the TSA with the rectangular corrugations showed the lowest operating frequency with a frequency band of 2.92–11.91 GHz. However, the antenna size was quite large compared to other compact TSAs in the literature. Six pairs of corrugations using slanted elliptical rectangular slots were used to miniaturize an antipodal TSA operating in the 4.5–50 GHz band, but the antenna size was also quite large [27].

Thirdly, the shape of the tapered slot was modified nonuniformly by varying the width of the tapered slot using a sinusoidal modulated Gaussian or a truncated Fourier series. A sinusoidal modulated Gaussian tapered slot with exponential slots on the ground conductor was proposed for a compact TSA operating from 2 GHz to 12 GHz [28]. A nonuniform tapered slot using a truncated Fourier series with cosine functions was introduced to design a miniaturized TSA with a frequency band of 2.9–13.55 GHz [29]. However, the tapered slot using a sinusoidal modulated Gaussian or a truncated Fourier series would also be very difficult to design in practice due to a complex structure.

To increase the gain of the miniaturized TSAs, directive materials using a parasitic metallic patch, a dielectric lens/cover with high relative permittivity, and a metamaterial loading/lens with periodic unit cells were added above the tapered slot aperture as a director [22]. Firstly, for the gain enhancement method using a parasitic metallic patch, various patch shapes, such as an elliptical, a diamond-shaped, or in multiple strips, were appended either inside the tapered slot or above the tapered slot [30,31,32,33]. Secondly, a dielectric lens/cover with different shapes, such as a half-elliptical shaped, a trapezoidal shaped, an exponential shaped, or a combination of two fan-shaped and a half-circular-shaped slots, was applied to enhance gain [34,35,36,37,38,39,40]. Thirdly, for a metamaterial loading/lens, different unit cell shapes, such as a meander-line, a modified split ring, a modified parallel line, or a rectangular patch, were used [41,42,43,44,45,46,47,48]. In addition to adding directive materials, methods of adding a horn structure and increasing the number of tapered slots were attempted to enhance gain of the compact TSAs. A pyramidal horn structure was combined with the TSA as an exterior surrounding structure to increase gain [49]. Two metallic plates can also be used instead of the horn structure with four plates to alleviate the cost and complexity of the fabrication [50,51]. A double-slot structure excited in uniform amplitude and phase by using a T-junction power divider was proposed to increase the gain of the TSA [52].

Meanwhile, RFID technologies, which use identification information transmitted from a tag attached to an object using electromagnetic waves to automatically recognize the attached object without contact using various frequencies, have been developed as a next-generation automatic recognition technology and have been widely used in various fields of everyday life, such as goods (asset) management, access control, food waste management, electronic passport, transportation card, highway electronic toll collection, clothing and book theft prevention, and medicine management [53,54]. To solve the problem of the high price for chip-based RFID tags, various studies have been conducted on chipless RFID tags [55]. Among the various methods realizing chipless RFID tags, a microwave resonator-based method has been actively investigated. The microwave resonator-based method can be divided into time domain, frequency domain, and hybrid methods depending on the domain in which electromagnetic wave signals are used [56,57]. Frequency domain methods can be divided into a retransmission-based method using transmitting and receiving antennas combined with resonators and a back scattering-based method using only resonators. As mentioned earlier, a compact wideband directive antenna with moderate gain is required for the retransmission-based method in order to receive the interrogation electromagnetic wave of the reader and transmit the spectral signature of the tag for identification (ID) or sensing information back to the reader [10,58].

In this paper, two design methods for a compact wideband TSA using fan-shaped and stepped structures for chipless RFID sensor tag applications were proposed. First, a miniaturization of the TSA by sequentially appending the fan-shaped structures using quarter circular slots, half circular slots, and half circular patches to the sides of the ground conductor was proposed. Next, to further miniaturize the TSA, the fan-shaped structures combined with the stepped structures were added to the sides of the ground conductor. The variations in the input reflection coefficient and gain of the TSA were systematically compared when the fan-shaped slots or patches were added at each stage during the design process. In addition, the operating characteristics of the final-designed compact wideband TSAs using the two miniaturization methods were analyzed using the surface current density distributions and radiation patterns at several frequencies in the band. The proposed compact wideband antenna with the slots combined with the fan-shaped and stepped structures was fabricated on an RF-35 substrate to compare with the simulation results. All the simulated results in this paper were obtained using CST Studio Suite (Dassault Systèmes Co., Vélizy-Villacoublay, France) [59].

## 2. Miniaturization Using Fan-Shaped Structures

**Figure 1:**
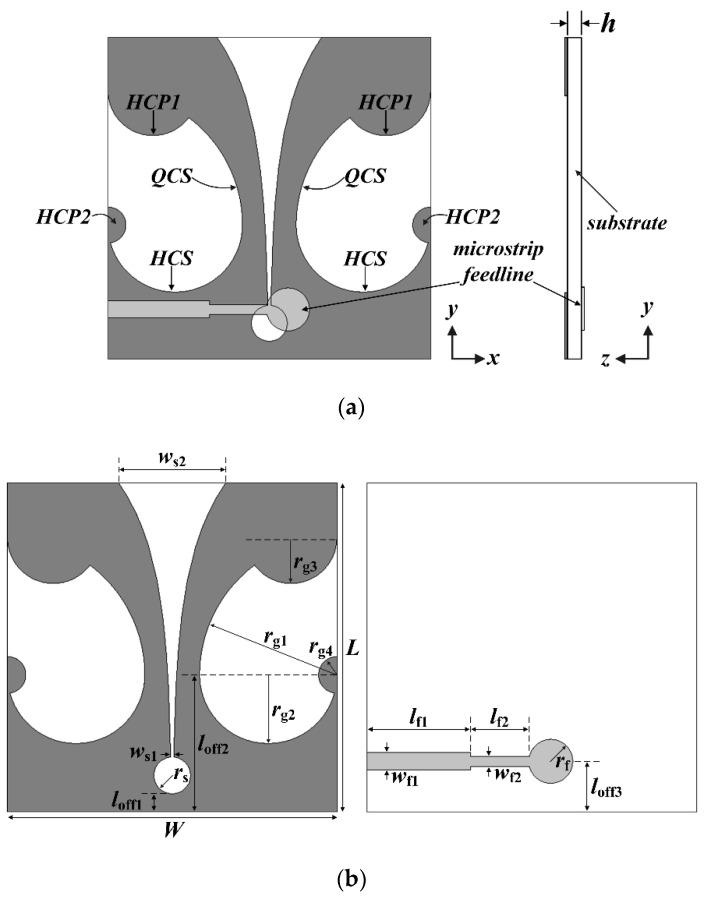
Geometries of the compact wideband TSA using fan-shaped structures (quarter circular slots (QCSs), half circular slots (HCSs), and half circular patches (HCP1s and HCP2s)): (**a**) whole structure and (**b**) top and bottom sides with design parameters.

The geometry of a compact wideband TSA using the fan-shaped structures is shown in Figure 1. On the top side of the substrate, a ground conductor consisting of an exponentially tapered slot terminated with a circular slot cavity and side slots using fan-shaped structures was printed, whereas a 50-ohm microstrip transmission line consisting of a circular termination stub and two-stage transmission lines of different widths was printed on the bottom side. An RF-35 substrate with a relative permittivity of *ε*_r_ = 3.5, a loss tangent of tan *δ* = 0.0018, and a thickness of *h* = 0.76 mm was used.

The tapered slot was designed using Equation (1). In the equation, *x* is the distance from the *x*-axis to the end of the exponential slot when the horizontal axis of the substrate is the *x*-axis and its origin is the center of the lower edge for the substrate. For instance, when *y* increases from 6 mm (*l*_off1_ + 2 × *r*_s_) to 36 mm (*L*), *x* increases from 0.16 mm to 5.85 mm exponentially, using the design parameters in Table 1.

(1) $$x=\pm \left\{c_{1}\exp\left(r_{1}\left(y-\left(l_{\mathrm{off}1}+2\times r_{s}\right)\right)\right)-c_{1}+\frac{w_{s1}}{2}\right\},\;\;\left(l_{\mathrm{off}1}+2\times r_{s}\right)\leq y\leq L$$

The quarter circular slots (QCSs) with a radius of *r*_g1_, the half circular slots (HCSs) with a radius of *r*_g2_, the half circular patches (HCP1s) with a radius of *r*_g3_, and the half circular patches (HCP2s) with a radius of *r*_g4_ were sequentially added on the sides of the ground conductor to miniaturize the TSA. We note that the QCSs are the fan-shaped structures with an angle of 90°, whereas the HCSs and HCPs are the fan-shaped structures with an angle of 180°. Table 1 shows the final design parameters of the compact wideband TSA using fan-shaped structures consisting of the QCSs, HCSs, HCP1s, and HCP2s.

**Figure 2:**
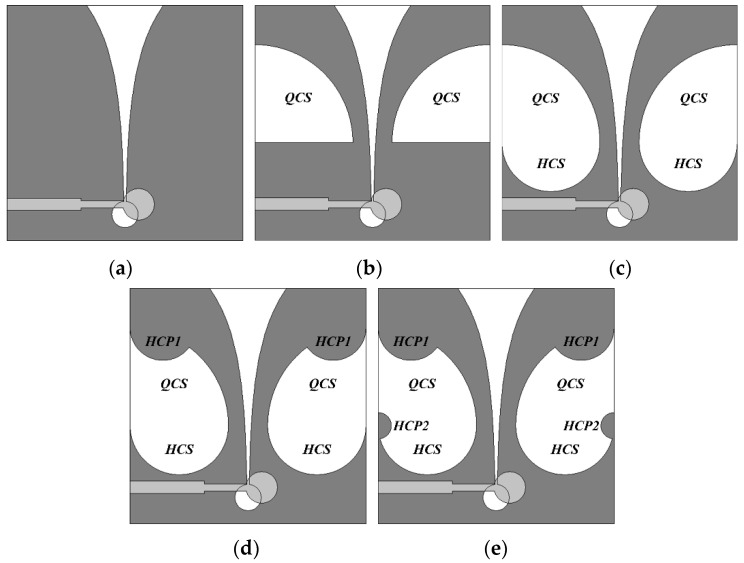
Design process of the compact wideband TSA using the fan-shaped structures: (**a**) conventional TSA without slots, (**b**) the TSA with the QCSs, (**c**) the TSA with the QCSs and HCSs, (**d**) the TSA with the QCSs, HCSs, and HCP1s, and (**e**) the TSA with the QCSs, HCSs, HCP1s, and HCP2s.

Figure 2 shows the antenna structures considered when the QCSs, HCSs, HCP1s, and HCP2s are added sequentially during the design process of the compact wideband TSA using the fan-shaped structures. Figure 2a is the geometry of a conventional TSA without the slots on the sides of the ground conductor, whereas Figure 2b shows the TSA with the QCSs with a radius of *r*_g1_ appended to the sides of the ground conductor. Figure 2c is the geometry of the TSA with the QCSs with a radius of *r*_g1_ and the HCSs with a radius of *r*_g2_, whereas Figure 2d shows the TSA with the QCSs with a radius of *r*_g1_, the HCSs with a radius of *r*_g2_, and the HCP1s with a radius of *r*_g3_. Figure 2e shows the final compact TSA with the QCSs with a radius of *r*_g1_, the HCSs with a radius of *r*_g2_, the HCP1s with a radius of *r*_g3_, and the HCP2s with a radius of *r*_g4_.

**Figure 3:**
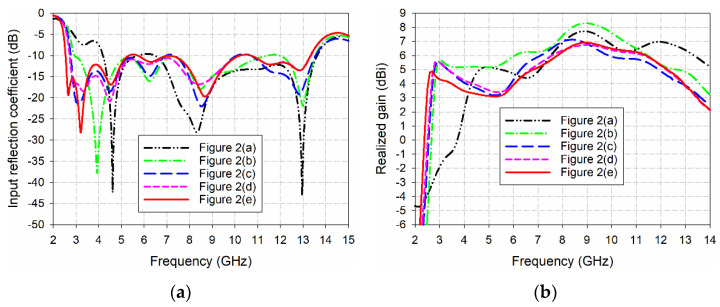
Performance comparison of the five antenna structures considered in the design process for the compact wideband TSA using the fan-shaped structures: (**a**) input reflection coefficient and (**b**) realized gain.

Figure 3 compares the input reflection coefficient and gain characteristics of the five antenna structures shown in Figure 2. In the case of the conventional TSA shown in Figure 2a without the slots on the sides of the ground conductor, the frequency band for a voltage standing wave ratio (VSWR) less than 2 was 4.157–13.670 GHz (106.7%), and gain in the band was 3.2–7.7 dBi. When the QCSs with a radius of *r*_g1_ = 16 mm were added to the sides of the ground conductor as shown in Figure 2b, the frequency band for a VSWR less than 2 was increased to 2.894–13.589 GHz (129.8%) and gain in the band was 4.0–8.3 dBi. Therefore, impedance bandwidth was increased by 23.1% and antenna size was reduced by 30.4%, compared to the conventional TSA in Figure 2a. Next, as the HCSs with a radius of *r*_g2_ = 8 mm were appended below the QCSs as shown in Figure 2c, the frequency band for a VSWR less than 2 was 2.754–13.605 GHz (132.7%), and gain in the band was 3.1–7.1 dBi. In this case, impedance bandwidth was increased by 26.0% and antenna size was reduced by 33.8%, compared to the conventional TSA. When the HCP1s with a radius of *r*_g3_ = 5 mm were added to the upper corners of the QCSs, as shown in Figure 2d, the frequency band for a VSWR less than 2 was 2.704–13.391 GHz (132.8%) and gain in the band was 3.1–6.8 dBi. Hence, impedance bandwidth was increased by 26.1% and antenna size was reduced by 35.0%, compared to the conventional TSA. Finally, when the HCP2s with a radius of *r*_g4_ = 2 mm were appended to the lower corners of the QCSs as shown in Figure 2e, the frequency band for a VSWR less than 2 was 2.530–13.379 GHz (136.4%) and gain in the band was 3.1–6.9 dBi. Therefore, impedance bandwidth of the compact wideband TSA with the QCSs, HCSs, HCP1s, and HCP2s was increased by 29.7% and antenna size was reduced by 39.1%, compared to the conventional TSA.

**Figure 4:**
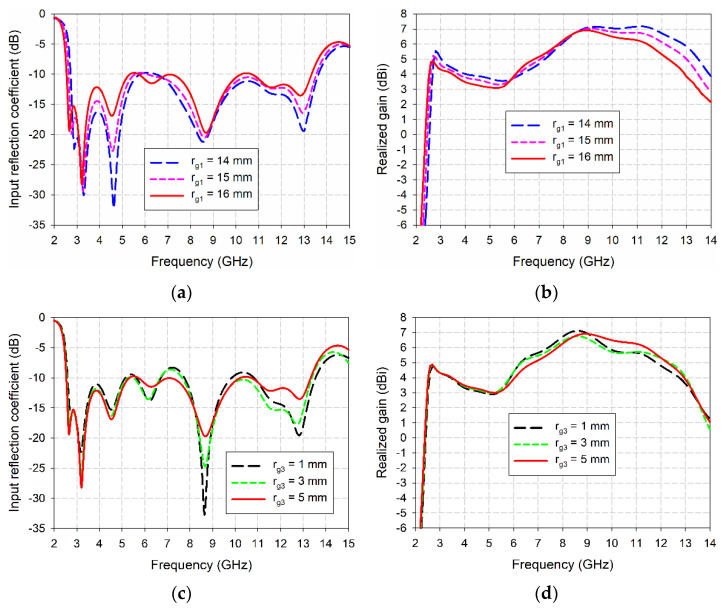
Effects of varying the radius *r*_g1_ of the QCSs and the radius *r*_g3_ of the HCP1s on the performance of the compact wideband TSA with fan-shaped structures: (**a**) input reflection coefficient for varying *r*_g1_, (**b**) realized gain for varying *r*_g1_, (**c**) input reflection coefficient for varying *r*_g3_, and (**d**) realized gain for varying *r*_g3_.

Figure 4 shows a comparison of the effects of varying the radius *r*_g1_ of the QCSs and the radius of *r*_g3_ of the HCP1s on the input reflection coefficient and realized gain characteristics of the compact wideband TSA with fan-shaped structures. For varying *r*_g1_, it was varied from 14 mm to 16 mm with a step of 1 mm and other design parameters were fixed at the values in Table 1. For varying *r*_g3_, it was varied from 1 mm to 5 mm with a step of 2 mm, and other design parameters were fixed at the values in Table 1. It turned out that the frequency band of the input reflection coefficient moved toward low frequency, and the bandwidth slightly increased as *r*_g1_ increased from 14 mm to 16 mm. However, gain in the band decreased slightly due to the effect of size reduction. For *r*_g1_ = 14 mm, the frequency band for a VSWR less than 2 was 2.708–13.687 GHz (133.9%), and gain in the band was 3.6–7.2 dBi. As *r*_g1_ increased to 15 mm, the frequency band for a VSWR less than 2 shifted toward low frequency, i.e., 2.616–13.586 GHz (135.4%), and gain in the band was 3.3–7.0 dBi. When *r*_g1_ increased further to 16 mm, the frequency band for a VSWR less than 2 moved further toward low frequency, i.e., 2.530–13.379 GHz (136.4%), and gain in the band was 3.1–6.9 dBi.

The effects of varying *r*_g3_ also showed a similar trend to the *r*_g1_ results with relatively smaller variations in the frequency bandwidth and gain characteristics. For *r*_g3_ = 1 mm, the frequency band for a VSWR less than 2 was 2.584–13.595 GHz (136.1%) with some deteriorated middle-frequency regions, and gain in the band was 2.2–7.1 dBi with reduced gain in the high-frequency region. As *r*_g3_ increased to 3 mm, the frequency band for a VSWR less than 2 shifted toward low frequency, i.e., 2.552–13.432 GHz (136.1%) with some deteriorated middle-frequency regions, and gain in the band was 2.6–6.8 dBi with reduced gain in the middle- and high-frequency regions. When *r*_g3_ increased further to 5 mm, the frequency band for a VSWR less than 2 moved further toward low frequency, i.e., 2.530–13.379 GHz (136.4%), and gain in the band was 3.1–6.9 dBi. Therefore, the value of *r*_g3_ needs to be carefully determined by looking at impedance matching and gain variations in the band.

**Figure 5:**
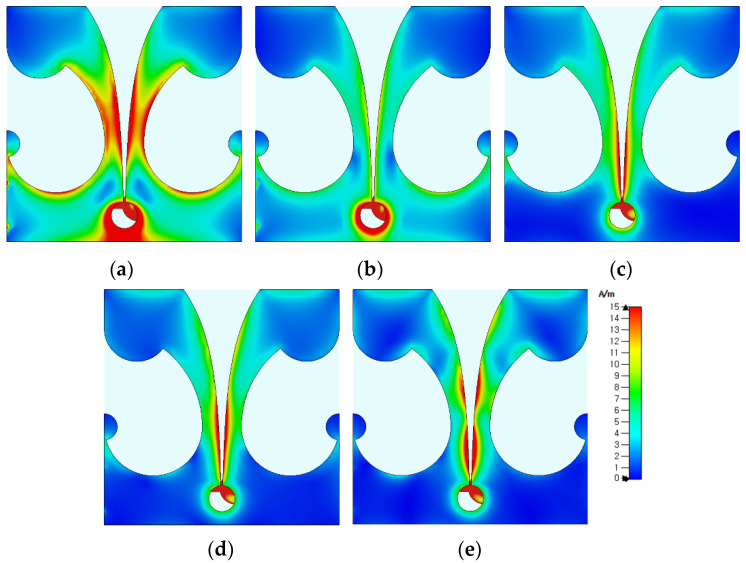
Surface current distributions of the compact wideband TSA using the fan-shaped structures at (**a**) 2.53 GHz, (**b**) 3.1 GHz, (**c**) 6 GHz, (**d**) 9 GHz, and (**e**) 12 GHz.

Figure 5 shows the simulated surface current distributions of the compact wideband TSA using the fan-shaped structures with *r*_g1_ = 16 mm at 2.53 GHz, 3.1 GHz, 6 GHz, 9 GHz, and 12 GHz. The surface currents at low frequencies, such as 2.53 GHz and 3.1 GHz, were distributed on the whole structure of the TSA, including the slots on the ground conductor and the tapered slot. As the frequency increased, the surface currents were mainly distributed on the tapered slot. For high frequencies at 9 GHz and 12 GHz, the length of the tapered slot is larger than one guided wavelength at these frequencies, and the sidelobe levels were increased.

**Figure 6:**
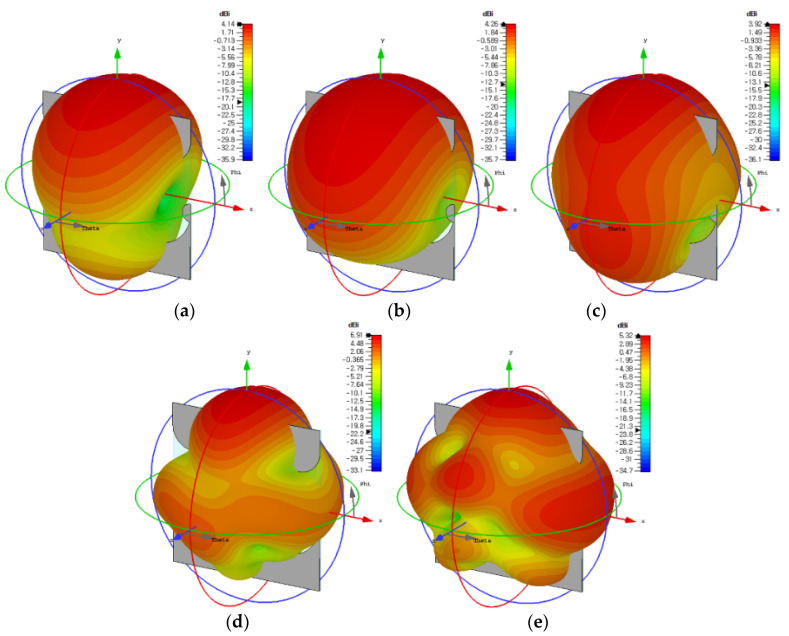
Three-dimensional radiation patterns of the compact wideband TSA using the fan-shaped structures at (**a**) 2.53 GHz, (**b**) 3.1 GHz, (**c**) 6 GHz, (**d**) 9 GHz, and (**e**) 12 GHz.

**Figure 7:**
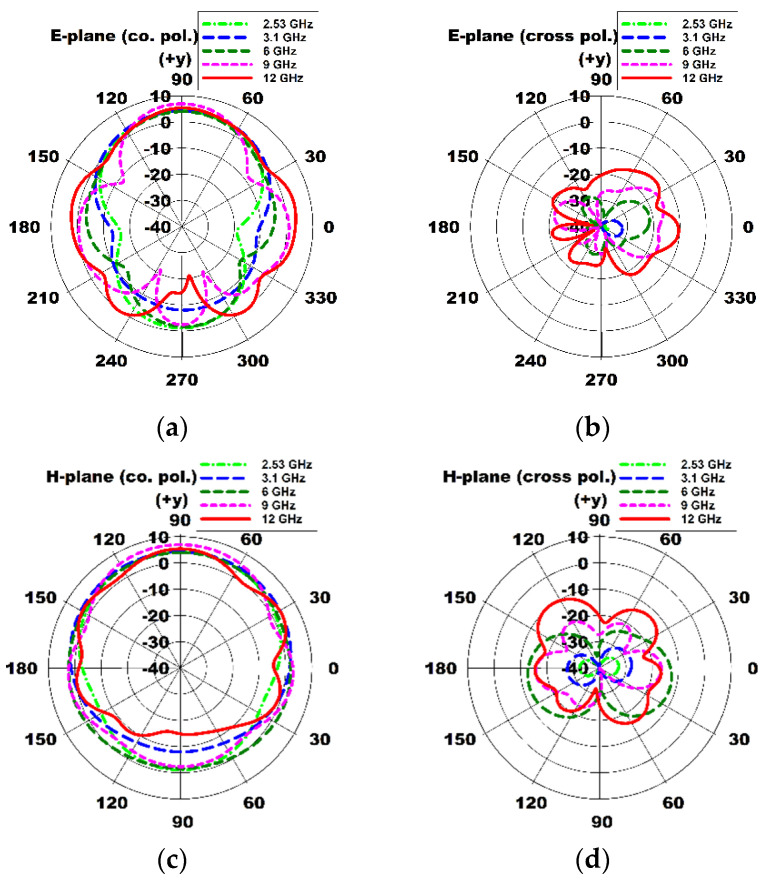
Comparison of E- and H-plane radiation patterns of the compact wideband TSA using the fan-shaped structures at 2.53 GHz, 3.1 GHz, 6 GHz, 9 GHz, and 12 GHz: (**a**) E-plane co-pol. (*x*-*y* plane), (**b**) E-plane cross-pol. (*x*-*y* plane), (**c**) H-plane co-pol. (*y*-*z* plane), and (**d**) H-plane cross-pol. (*y*-*z* plane).

Three-dimensional radiation patterns of the compact wideband TSA using the fan-shaped structures at 2.53 GHz, 3.1 GHz, 6 GHz, 9 GHz, and 12 GHz are compared in Figure 6, whereas Figure 7 shows the co-pol. (polarization) and cross-pol. radiation patterns on the E-plane (*x*-*y* plane) and H-plane (*y*-*z* plane) at 2.53 GHz, 3.1 GHz, 6 GHz, 9 GHz, and 12 GHz. It can be seen that the sidelobe and cross-pol. levels increased as the frequency increased.

## 3. Miniaturization Using Fan-Shaped and Stepped Structures

**Figure 8:**
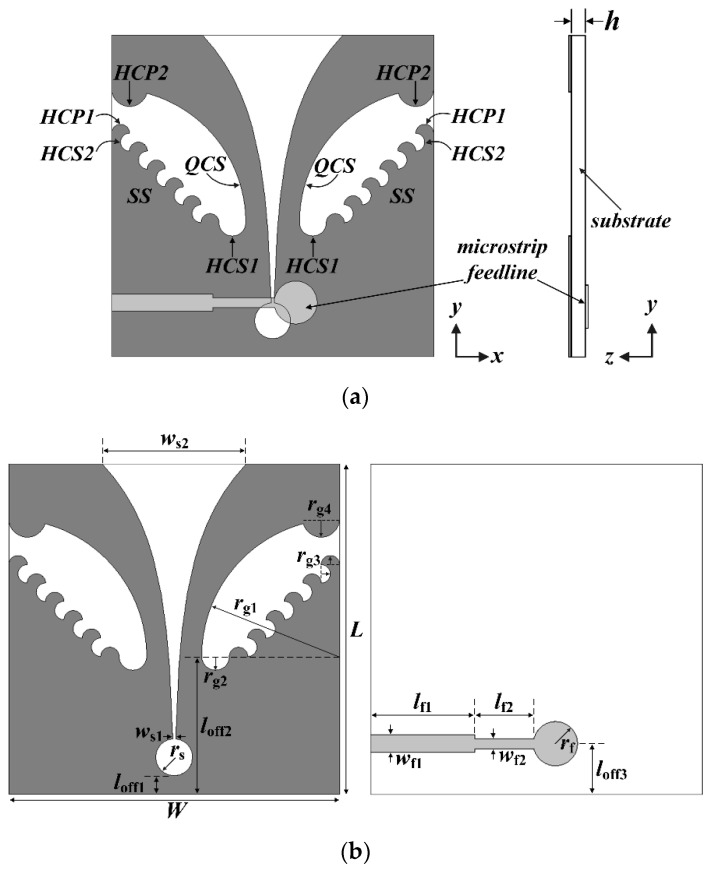
Geometries of the compact wideband TSA using the fan-shaped and stepped structures: (**a**) whole structure and (**b**) top and bottom sides with design parameters.

This section deals with a method of combining the fan-shaped structures and a stepped structure to further decrease the size of the TSA. An attempt to miniaturize the TSA by combining the quarter circular slots and a stepped structure has been studied in [16]. For further miniaturization, we investigated appending the half circular slots and half circular patches on the stepped structure. Figure 8 shows the geometry of the compact wideband TSA using a combination of the fan-shaped and stepped structures.

The configuration of the compact wideband TSA using the fan-shaped and stepped structures is similar to that of the compact wideband TSA using the fan-shaped structures. For the miniaturization of the TSA, the quarter circular slots (QCSs) with a radius of *r*_g1_, the stepped structures (SSs) with five steps, the half circular slots (HCS1s) with a radius of *r*_g2_, the half circular patches (HCP1s) and half circular slots (HCS2s) with a radius of *r*_g3_, and the half circular patches (HCP2s) with a radius of *r*_g4_ were sequentially appended on the sides of the ground conductor. Table 2 shows the final design parameters of the compact wideband TSA using the fan-shaped and stepped structures consisting of the quarter circular slots, stepped structures, half circular slots, and half circular patches. Note that *c*_1_ slightly increased to 0.23 compared to that of the compact wideband TSA using the fan-shaped structures.

**Figure 9:**
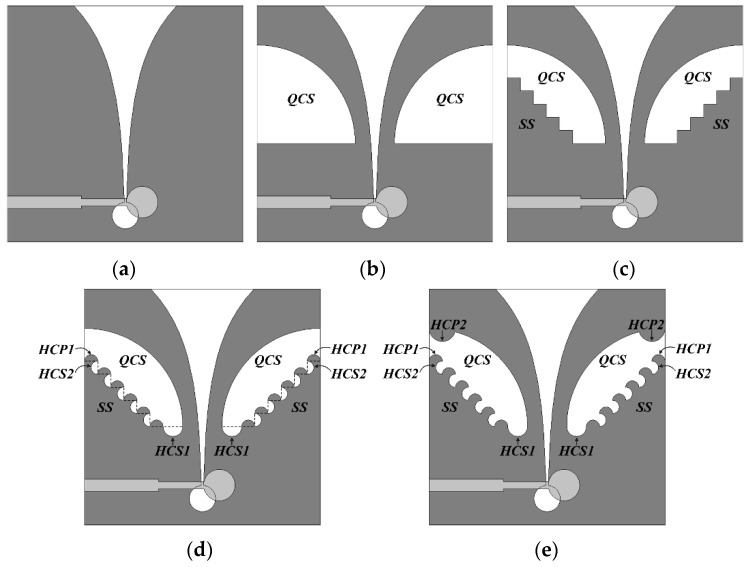
Design process of the compact wideband TSA using the fan-shaped and stepped structures: (**a**) conventional TSA without slots; (**b**) the TSA with the QCSs; (**c**) the TSA with the QCSs and SSs; (**d**) the TSA with the QCSs, SSs, HCS1s, HCS2s, and HCP1s; and (**e**) the TSA with the QCSs, SSs, HCS1s, HCS2s, HCP1s, and HCP2s.

The antenna structures considered when the QCSs, SSs, HCS1s, HCP1s, HCS2s, and HCP2s are added sequentially during the design process of the compact wideband TSA using the fan-shaped and stepped structures are shown in Figure 9. Figure 9a is the geometry of a conventional TSA without the slots on the sides of the ground conductor, whereas Figure 9b shows the TSA with the QCSs with a radius of *r*_g1_ appended to the sides of the ground conductor. Figure 9c is the geometry of the TSA with the QCSs with a radius of *r*_g1_ and the SSs with five steps, whereas Figure 9d shows the TSA with the QCSs with a radius of *r*_g1_, the SSs with five steps, the HCS1s with a radius of *r*_g2_, and the HCP1s and HCS2s with a radius of *r*_g3_. Figure 9e shows the final compact TSA with the QCSs with a radius of *r*_g1_, the SSs with five steps, the HCS1s with a radius of *r*_g2_, the HCP1s and HCS2s with a radius of *r*_g3_, and the HCP2s with a radius of *r*_g4_.

**Figure 10:**
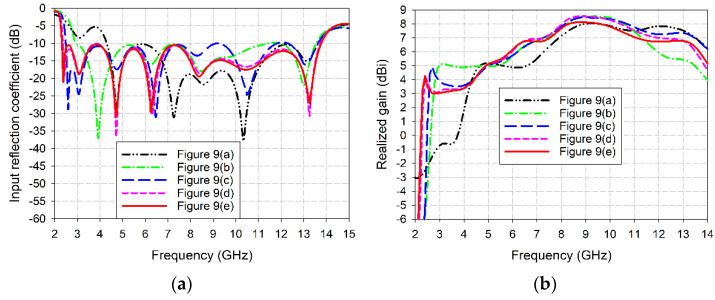
Performance comparison of the five antenna structures considered in the design process for the compact wideband TSA using the fan-shaped and stepped structures: (**a**) input reflection coefficient and (**b**) realized gain.

The performances such as input reflection coefficient and gain characteristics of the five antenna structures shown in Figure 9 were compared in Figure 10. The frequency band of the conventional TSA in Figure 9a without the slots for a VSWR less than 2 was 4.276–13.691 GHz (104.8%), and gain in the band was 3.7–8.0 dBi. The frequency band for a VSWR less than 2 was increased to 3.178–13.711 GHz (124.7%) and gain in the band was 5.1–8.5 dBi when the QCSs with a radius of *r*_g1_ = 16 mm were added to the sides of the ground conductor as shown in Figure 9b. In this case, impedance bandwidth was increased by 19.9% and antenna size was reduced by 25.7%, compared to the conventional TSA in Figure 9a. Next, as the SSs with five steps having a step length of 2 mm (2 × *r*_g3_) were appended in the center of the QCSs as shown in Figure 9c, the frequency band for a VSWR less than 2 was 2.483–13.671 GHz (138.5%) and gain in the band was 3.5–8.5 dBi. Hence, impedance bandwidth was increased by 33.7.0% and antenna size was reduced by 41.9%, compared to the conventional TSA. When the HCS1s with a radius of *r*_g2_ = 2 mm were added to the lower corners of the QCSs, and the HCP1s and HCS2s with a radius of *r*_g3_ = 1 mm were added in the steps of the SSs, as shown in Figure 9d, the frequency band for a VSWR less than 2 was 2.360–13.767 GHz (141.5%) and gain in the band was 3.1–8.6 dBi. In this case, impedance bandwidth was increased by 36.7% and antenna size was reduced by 44.8%, compared to the conventional TSA.

Finally, when the HCP2s with a radius of *r*_g4_ = 2 mm were appended to the upper corners of the QCSs as shown in Figure 9e, the frequency band for a VSWR less than 2 was 2.313–13.805 GHz (142.6%) and gain in the band was 3.0–8.1 dBi. Therefore, impedance bandwidth of the compact wideband TSA with the QCSs, SSs, HCS1s, HCP1s, HCP2s, and HCP2s was increased by 37.8% and antenna size was reduced by 45.9%, compared to the conventional TSA. In addition, when compared to the TSA with the QCSs and SSs in Figure 9c, impedance bandwidth was increased by 4.1% and antenna size was reduced by 6.9%.

**Figure 11:**
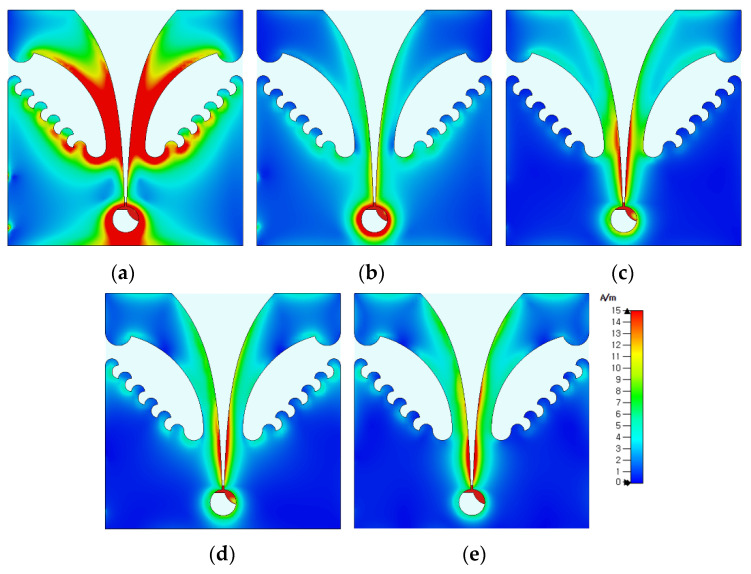
Comparison of the surface current distributions of the compact wideband TSA using the fan-shaped and stepped structures at (**a**) 2.313 GHz, (**b**) 3.1 GHz, (**c**) 6 GHz, (**d**) 9 GHz, and (**e**) 12 GHz.

Figure 11 shows the simulated surface current distributions of the compact wideband TSA using the fan-shaped and stepped structures at 2.313 GHz, 3.1 GHz, 6 GHz, 9 GHz, and 12 GHz. Similar to the compact wideband TSA using the fan-shaped structures only, the surface currents at low frequencies such as 2.313 GHz and 3.1 GHz were distributed on the whole structure of the TSA, including the slots on the ground conductor and the tapered slot. As the frequency increased, the surface currents were mainly distributed on the tapered slot.

**Figure 12:**
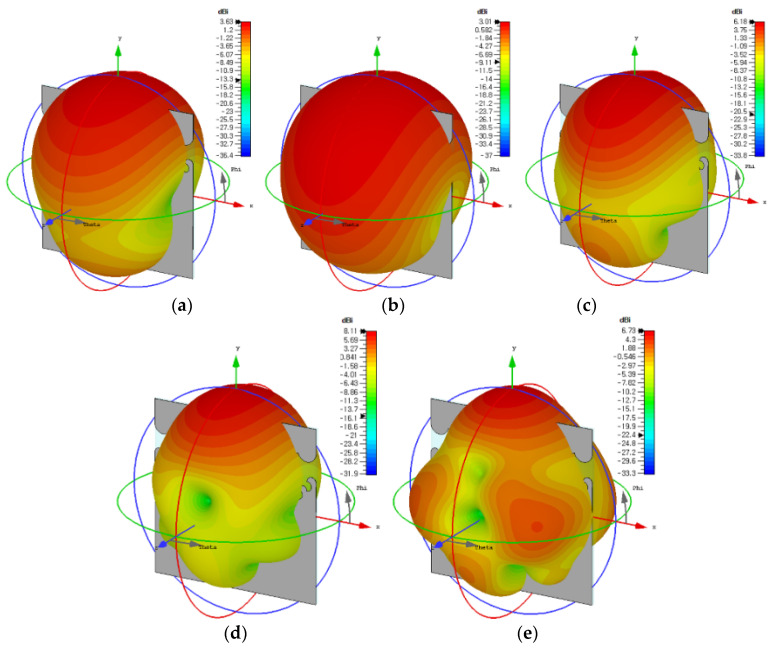
Three-dimensional radiation patterns of the compact wideband TSA using the fan-shaped and stepped structures at (**a**) 2.313 GHz, (**b**) 3.1 GHz, (**c**) 6 GHz, (**d**) 9 GHz, and (**e**) 12 GHz.

**Figure 13:**
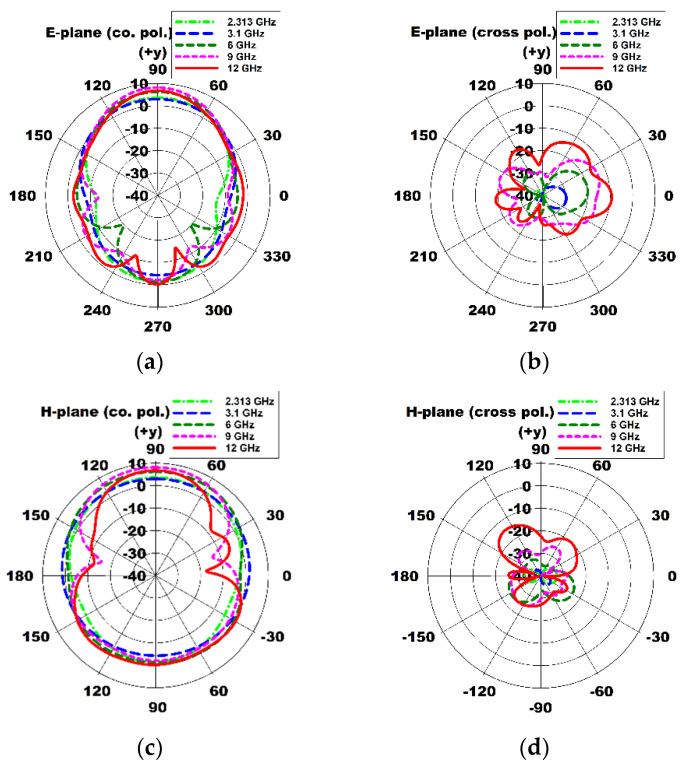
Comparison of E- and H-plane radiation patterns of the compact wideband TSA using the fan-shaped and stepped structures at 2.313 GHz, 3.1 GHz, 6 GHz, 9 GHz, and 12 GHz: (**a**) E-plane co-pol. (*x*-*y* plane), (**b**) E-plane cross-pol. (*x*-*y* plane), (**c**) H-plane co-pol. (*y*-*z* plane), and (**d**) H-plane cross-pol. (*y*-*z* plane).

Three-dimensional radiation patterns of the compact wideband TSA using the fan-shaped and stepped structures at 2.313 GHz, 3.1 GHz, 6 GHz, 9 GHz, and 12 GHz are compared in Figure 12. The co-pol. and cross-pol. radiation patterns in the E-plane (*x*-*y* plane) and H-plane (*y*-*z* plane) at 2.313 GHz, 3.1 GHz, 6 GHz, 9 GHz, and 12 GHz are shown in Figure 13. We note that the sidelobe levels at high frequencies such as 9 GHz and 12 GHz decreased compared to those of the compact wideband TSA using the fan-shaped structures only. It is conjectured that the ground slots using the fan-shaped and stepped structures were finished far above the feed line compared to when only the fan-shaped structures were used, and this lowered the sidelobe levels.

## 4. Experiment Results and Discussion

**Figure 14:**
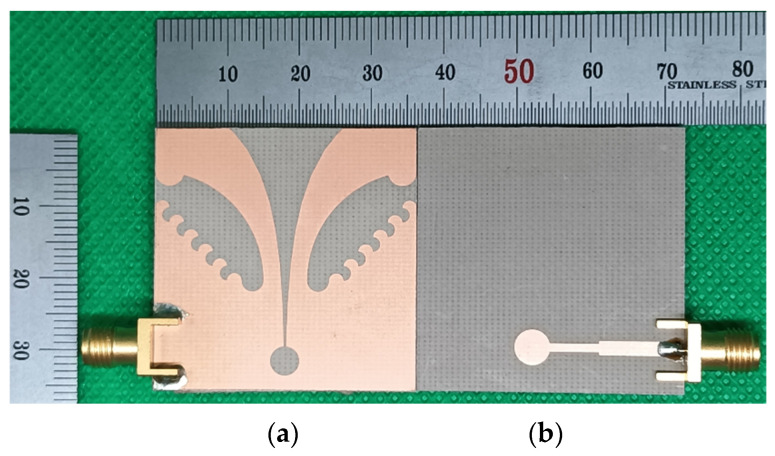
Photographs of the fabricated compact wideband TSA using the fan-shaped and stepped structures: (**a**) top view and (**b**) bottom view.

**Figure 15:**
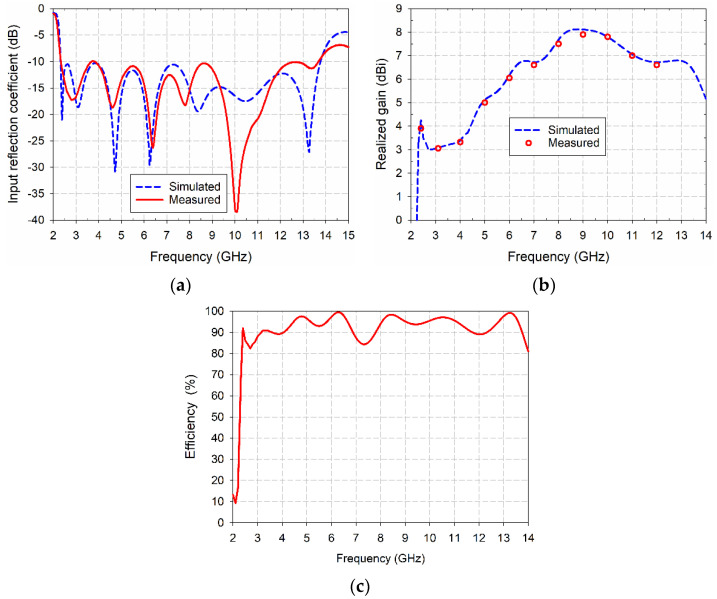
Measured performance of the fabricated compact wideband TSA using the fan-shaped and stepped structures: (**a**) input reflection coefficient, (**b**) realized gain, and (**c**) simulated efficiency.

A prototype of the compact wideband TSA using the fan-shaped and stepped structures was fabricated on an RF-35 substrate to validate the simulation results, as shown in Figure 14. Input reflection coefficient characteristic of the fabricated compact wideband TSA using the fan-shaped and stepped structures was measured using an Agilent N5230 (Agilent Technologies Inc., Santa Clara, CA, USA) vector network analyzer. The measured frequency band for a VSWR less than 2 was 2.320–13.745 GHz (142.2%), whereas it was 2.313–13.805 GHz (142.6%) for the simulated result, as shown in Figure 15a. The lower limit of the measured frequency band slightly increased, and the upper limit decreased. Therefore, the measured frequency band slightly decreased compared to the simulated one.

Measured gain of the fabricated compact wideband TSA using the fan-shaped and stepped structures was compared in Figure 15b. It ranged from 3.1 dBi to 7.9 dBi, which is similar to the simulated one. A simulated total efficiency of the fabricated compact wideband TSA using the fan-shaped and stepped structures is plotted in Figure 15c. Total efficiency ranged from 80% to 99.5% in the band.

**Figure 16:**
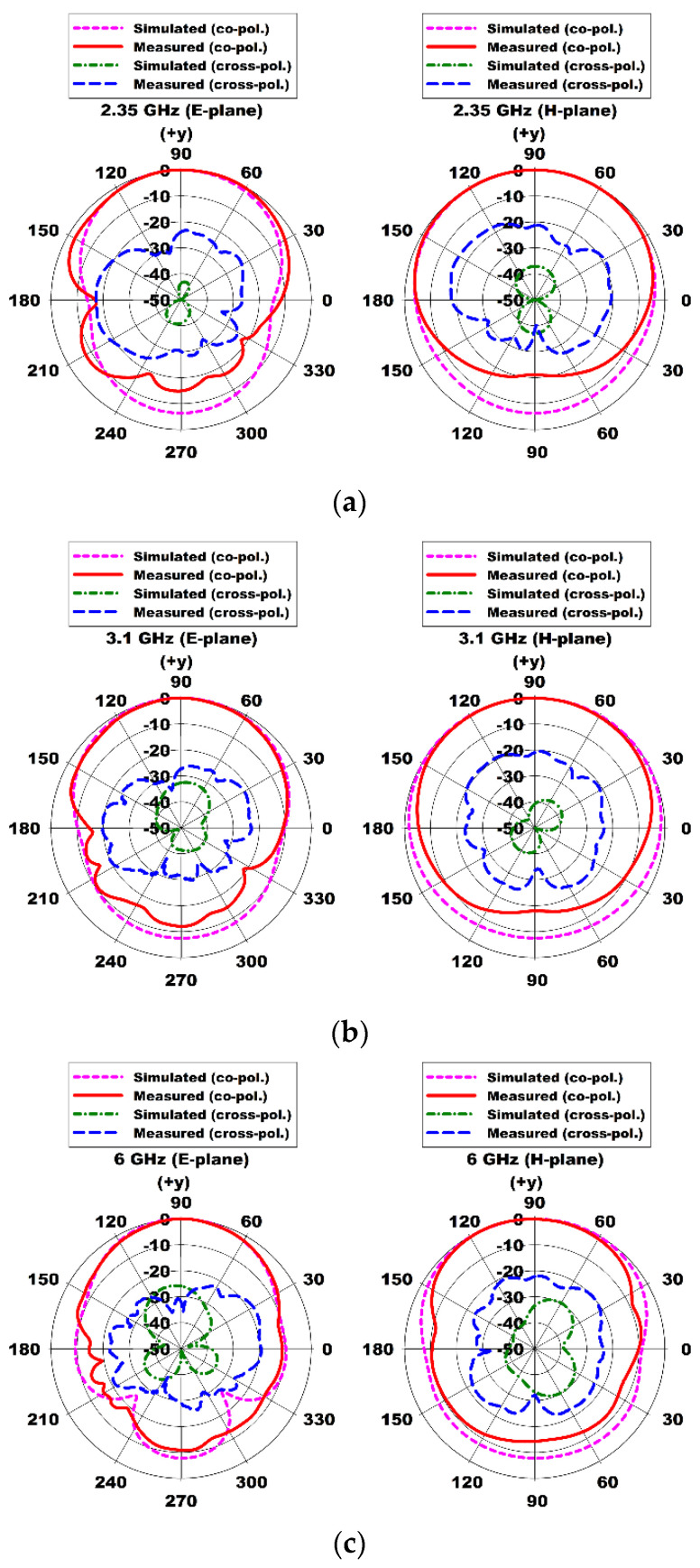

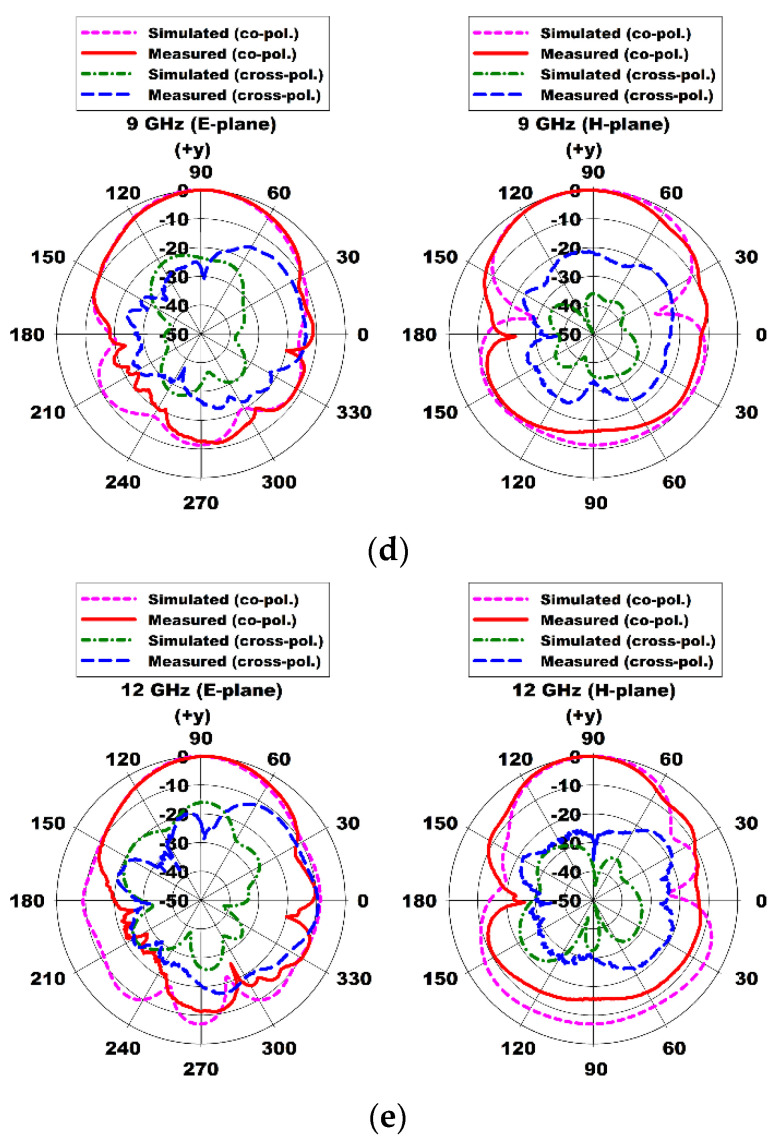
Measured radiation patterns in the E- and H-planes of the compact wideband TSA using the fan-shaped and stepped structures at (**a**) 2.35 GHz, (**b**) 3.1 GHz, (**c**) 6 GHz, (**d**) 9 GHz, and (**e**) 12 GHz.

Figure 16 shows the comparison of the measured and simulated radiation patterns in the E- and H-planes at 2.35 GHz, 3.1 GHz, 6 GHz, 9 GHz, and 12 GHz. The measured and simulated radiation patterns agreed well with each other for both planes, but the measured cross pol. levels were increased due to the effects of the coaxial cable used for measurement. Cross pol. levels can be suppressed by using a double-layer structure instead of a single-layer one [20] or an antipodal structure with increased height.

**Figure 17:**
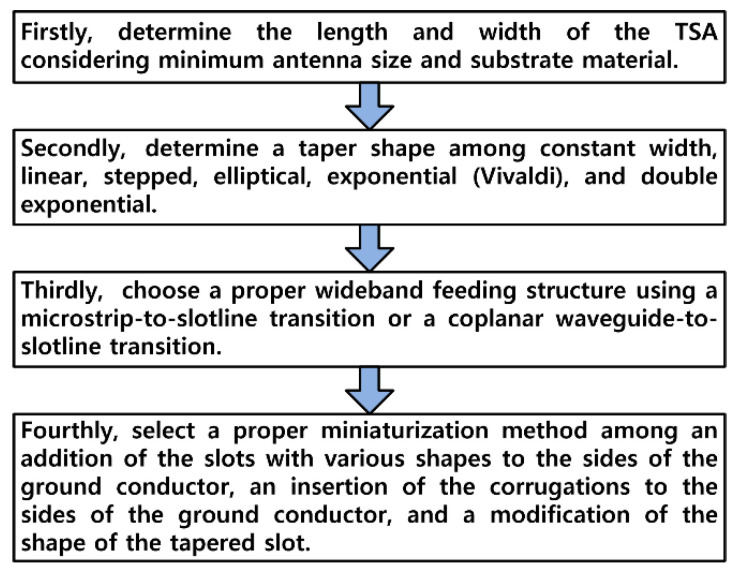
General design procedure of compact wideband TSA.

Table 3 compares the dimensions and performances of the proposed compact wideband TSA using the fan-shaped and stepped structures with other compact wideband TSAs in the literature. Electrical dimensions of the antennas were calculated using the free-space wavelength of the lower limit frequency (λ_L_) for each antenna. We can see that the frequency bandwidth of the proposed compact wideband TSA using the fan-shaped and stepped structures is the largest among the antennas except [14,17,19,24,27], but the electrical dimensions considering the lower limit frequency are smaller than these. In addition, the electrical dimensions of the proposed TSA are the smallest among the antennas except [14,16,23,29], but the lowest gain is larger compared to them. In this regard, tradeoff between the lowest gain in the band and impedance frequency bandwidth must be necessary for designing the compact TSA considering minimum antenna size. Figure 17 shows the general design procedure of the compact wideband TSA. Firstly, determine the length and width of the TSA considering minimum antenna size and substrate material. The longer the TSA’s length, the lower the lowest limit frequency and the wider the frequency bandwidth with increased gain. Secondly, determine a taper shape among constant width, linear, stepped, elliptical, exponential (Vivaldi), and double exponential. Thirdly, choose a proper wideband feeding structure, such as a microstrip-to-slotline transition or a coplanar waveguide-to-slotline transition. The shapes and sizes of the open conductor stub and slot cavity should be optimized for wideband operation. Fourthly, select a proper miniaturization method among an addition of the slots with various shapes to the sides of the ground conductor, an insertion of the corrugations to the sides of the ground conductor, and a modification of the shape of the tapered slot.

## 5. Conclusions

We proposed two miniaturization methods for a compact wideband TSA either using the fan-shaped structures only or using a combination of the fan-shaped and stepped structures. The first miniaturization method was an addition of the fan-shaped structures to the sides of the ground conductor of the TSA. The quarter circular slots, the half circular slots, and the half circular patches were sequentially appended to the sides of the ground conductor, and their effects on the input reflection coefficient and gain characteristics of the TSA were compared. The frequency band of the compact wideband TSA with the fan-shaped structures using the QCSs, HCSs, HCP1s, and HCP2s for a VSWR less than 2 was 2.530–13.379 GHz (136.4%) and gain in the band was 3.1–6.9 dBi. In this case, impedance bandwidth was increased by 29.7% and antenna size was reduced by 39.1%, compared to the conventional TSA. The second miniaturization method to further reduce the antenna size was a combination of the fan-shaped and stepped structures. Similar to the first miniaturization method, the quarter circular slots, the stepped structures, the half circular slots, and the half circular patches were sequentially appended to the sides of the ground conductor, and their effects on the input reflection coefficient and gain characteristics of the TSA were compared. The frequency band of the compact wideband TSA with the fan-shaped and stepped structures using the QCSs, SSs, HCS1s, HCP1s, HCP2s, and HCP2s for a VSWR less than 2 was 2.313–13.805 GHz (142.6%) and gain in the band was 3.0–8.1 dBi. Impedance bandwidth was increased by 37.8% and antenna size was reduced by 45.9%, compared to the conventional TSA. The operating characteristics of the compact wideband TSAs using the two miniaturization methods were also analyzed using the surface current density distributions and radiation patterns at several frequencies in the band. It turned out that the sidelobe levels at high frequencies such as 9 GHz and 12 GHz of the compact wideband TSA with the second miniaturization method using the fan-shaped and stepped structures decreased compared to those of the compact wideband TSA with the first miniaturization method using the fan-shaped structures only.

A prototype of the compact wideband TSA using the fan-shaped and stepped structures was fabricated on an RF-35 substrate to validate the simulation results. The measured frequency band for a VSWR less than 2 was 2.320–13.745 GHz (142.2%), and the measured frequency band slightly decreased compared to the simulated result. Measured gain ranged from 3.1 dBi to 7.9 dBi, which is similar to the simulated one. The measured and simulated radiation patterns agreed well with each other for both E- and H-planes.

The proposed compact wideband TSAs using the fan-shaped and stepped structures can be used for designing compact wideband directive antennas for retransmission-based chipless RFID sensor tag applications in real-life scenarios. They might also be used for UWB radar and imaging applications, such as the through-wall human detection and identification of movements, human vital signal monitoring, and breast cancer detection imaging system.

As part of future work, we plan to study a new method with a wider bandwidth and miniaturization rate by combining the various miniaturization methods analyzed in this paper.

## Figures and Tables

**Table 1:** Design parameters of the compact wideband TSA using fan-shaped structures.

Parameter	Value (mm)	Parameter	Value (mm)
*W*	36	*w*_f2_	1.1
*L*	36	*r*_f_	2.4
*w*_s1_	5	*r*_g1_	16
*w*_s2_	11.7	*r*_g2_	8
*l*_off1_	2	*r*_g3_	5
*l*_off2_	15	*r*_g4_	2
*l*_off3_	5.6	*r*_s_	2
*l*_f1_	11.3	*c*_1_	0.17
*l*_f2_	6.5	*r*_1_	0.118
*w*_f1_	1.9	*h*	0.76

**Table 2:** Design parameters of the compact wideband TSA using the fan-shaped and stepped structures.

Parameter	Value (mm)	Parameter	Value (mm)
*W*	36	*w*_f2_	1.1
*L*	36	*r*_f_	2.4
*w*_s1_	5	*r*_g1_	16
*w*_s2_	11.7	*r*_g2_	2
*l*_off1_	2	*r*_g3_	1
*l*_off2_	15	*r*_g4_	2
*l*_off3_	6.1	*r*_s_	2
*l*_f1_	11.3	*c*_1_	0.23
*l*_f2_	7	*r*_1_	0.118
*w*_f1_	1.9	*h*	0.76

**Table 3:** Comparison of the dimensions and performances of the compact wideband TSA using the fan-shaped and stepped structures with other compact wideband TSAs in the literature.

References	Miniaturization Methods	Physical Dimensions (mm^3^)	Electrical Dimensions (λ_L_^3^)	Bandwidth for VSWR < 2 (GHz)	Gain (dBi)
[13]	Triangular slot and corrugations	35 × 36 × 0.8	0.362 × 0.372 × 0.008	3.1–10.6 (109.5%)	2–8.5
[14]	Anti-spiral shape and lumped resistors	63.5 × 53 × -	0.254 × 0.212 × -	1.2–9.8 (156.4%)	-
[15]	Quarter circular slots and nonuniform corrugations	37 × 34 × 0.8	0.382 × 0.351 × 0.008	3.1–10.6 (109.5%)	1.5–8.1
[16]	Quarter circular slots and stepped structures	36 × 30 × 1.0	0.282 × 0.235 × 0.008	2.35–11.0 (129.6%)	3.0–7.8
[17]	Half circular slots	44 × 62 × 0.254	0.66 × 0.93 × 0.004	4.5–50.0 (167.0%)	3.0–12.0
[18]	Two pairs of eye-shaped slots	36 × 36 × 0.8	0.36 × 0.36 × 0.008	3.0–12.8 (124.1%)	3.7–8.3
[19]	Resonant cavities consisting of an eye-shaped slot and a circular slot	150 × 258 × 0.8	0.25 × 0.43 × 0.001	0.5–6.0 (169.2%)	0.8–8.0
[20]	Modified exponential slots	36 × 32 × 2	0.3 × 0.267 × 0.017	2.5–11.0 (125.9%)	3.5–8.0
[21]	Third generation of Koch fractal curves and circular slots	36 × 60 × 0.64	0.584 × 0.974 × 0.01	4.87–11.0 (77.3%)	-
[22]	Hook-shaped slots	30 × 32 × 0.8	0.283 × 0.302 × 0.008	2.83–11.31 (119.9%)	3.2–7.5
[23]	Bent Vivaldi elements	48 × 48 × 22.5	0.256 × 0.256 × 0.12	1.6–2.9 (57.8%)	1.7–5.7 (dBic)
[24]	Four cascaded circular cavity structures based on several circular holes in tandem with different curvatures	195 × 244.2 × 1.57	0.295 × 0.366 × 0.0024	0.45–10 (182.8%)	−1–10.8
[25]	Corrugations using three pairs of trapezoidal slots	35 × 30 × 1	0.35 × 0.3 × 0.01	3.0–11.4 (116.7%)	3.0–7.4
[26]	42 pairs of rectangular corrugations	50 × 100 × 0.8	0.487 × 0.973 × 0.008	2.92–11.91 (121.2%)	3.9–10.5
[27]	6 pairs of corrugations using slanted elliptical rectangular slots	30 × 40 × 0.51	0.45 × 0.6 × 0.008	4.5–50 (167.0%)	3.0–10.0
[28]	Sinusoidal modulated Gaussian tapered slot	50 × 56 × 0.8	0.333 × 0.373 × 0.005	2.0–12.0 (142.9%)	1.5–5.2
[29]	Nonuniform tapered slot using a truncated Fourier series with cosine functions	30 × 20.3 × 0.8	0.288 × 0.196 × 0.008	2.9–13.55 (129.5%)	1.8–6.9
This Work	Fan-shaped and stepped structures	36 × 36 × 0.76	0.278 × 0.278 × 0.006	2.320–13.745 (142.2%)	3.1–7.9

## Data Availability

Data are contained within the article.
